# Why Are We Not a Healthy People?

**Published:** 1859-12

**Authors:** Charles H. Bass

**Affiliations:** Assistant Physician to the Georgia State Lunatic Asylum, Milledgeville, Ga.


### ARTICLE II.

*Why are we not a Healthy People?* By Charles H. Bass, M. D., Assistant Physician to the Georgia State Lunatic Asylum, Milledgeville, Ga.

It is a surprising and lamentable fact that Americans are not a healthy people. Disguise it as we may, it is a fact so patent that every traveler speaks of the sallow faced men and the nervous women of America. We, to whom nature has given a country superior to every other, with a varied, but temperate climate, with a freedom unknown in other lands, with a more equal distribution of wealth, and with a good mean rate of intelligence, we ought to be the healthiest nation on the globe. Yet, even England, foggy England, with her cold, damp climate, with her oppressive taxes, with her ale-drinking habits, has a higher rate of health than we have. It is true, that in Great Britain there are many thousand paupers, in the lowest possible condition of health, physical and mental, degraded in every respect, but except this class, and the brawny arms, and bluff, ruddy countenance of John Bull, shows off Brother Jonathan's long frame and careworn face, greatly to the latter's disadvantage. Indeed a comparison of the people of almost any

European country, those of them removed from want, with our people, will give the same result.

I propose to point out *some* of the causes of ill health peculiar to our people. A comparison of our climate with that of the countries of Europe, will result favorably to America. Our area is so great that we have a more varied climate than almost any other nation, but the Northern portion of the United States has a healthful, invigorating atmosphere, not so cold as the North of Europe, or more likely to produce disease; while at the South, we have a genial climate, free from the fatal diseases of cold regions, while the destructive fevers of warmer regions are confined to circumscribed localities. Our people are not crowded and pressed for room, as in some parts of Europe, and our institutions are for the most part, such as conduce to health and happiness. Moderate labor will always procure comfortable food and clothing. Industry and prudence will always prevent suffering and want. But our very form of government entails upon us ill health and suffering peculiar to itself; it at least conduces to the formation of habits, which are destructive to health and happiness. Our freedom tends to self-indulgence and excess. To be happy, contented and healthy, the appetites and passions must be governed; immoderate and ambitious desires must be restrained.

In our country the highest social and political position is in the reach of the man of energy. This imparts a restlessness to the American character, and a discontented spirit to the American people, at once unhealthy in its effects and destructive in its tendencies.

Our form of government gives rise to loose family government, and a free and untrammelled press, gives currency to opinions and sentiments often loose in their character, and detrimental in their effects.

While the press is the mouthpiece of public opinion, it has more influence than any other agency in the formation of public opinion. The political strifes which annually convulse the country, so arouse much contention, and the discussions of the press are conducted with so much acrimony, that public sentiment and taste are perverted. The baser passions of our nature are pandered to, a desire for excitement is generated, and a taste for strife and scandal is encouraged.

After the political campaign is over, these tastes are gratified by the recital of murders and robberies and intrigue. Minute details of criminal prosecutions, the mention of which should mantle the cheek of gentlemen, are given in our public prints which females and children read. Highly wrought tales of murder and revenge, of jealousy and intrigue, are spread broadcast through the land. This license of the press would indeed be ruinous were it not for the power of religion, the influence of the pulpit, the influence of the religious press, and the press controlled by religious principle.

These excitements are very unhealthy and dangerous in themselves and ruinous in their effects. The brain, acted upon by such causes is strained until it loses tone and vigor, and is rendered unfit to bear the trials to which our shifting fortunes are liable. Habits of dissipation are formed. Opium and tobacco are resorted to for their stimulating effects. Ardent spirits, that great bane of human happiness, is indulged in for the same reason. These excitements, and these habits, cause all manner of disease, and if the worst effects are not produced in the individual, a miserable constitution and a highly sensative nervous organization, is imparted to the unhappy offspring.

After the laws of nature have thus been disregarded, any shock, the loss of fortune or of friends, drags reason from her throne, and the brain, once the seat of lofty thought and high souled aspiration, is now busied with the wild imaginings of the lunatic, or sunk to the condition of drivelling idiocy.

Here it might be well to refer more particulary, to those habits of dissipation which produce such widespread wreck and ruin. Who can estimate the misery alcohol has caused.

Temperance orators and the temperance press have so often painted its revolting and hideous effects, that the picture has grown so familiar as to cease to startle, if not to interest the public. It is sickening and disheartening to the philanthropist to look out upon the desolation caused by alcohol. Alas, the disease upon the body politic, is of that festering, reproducing character, which if not skillfully managed will annually grow in extent and increase in virulence.

Wine-drinking fathers have dram-drinking sons, who fall into drunkards' graves, leaving children to sink lower into the depths of degradation than themselves. If, with us, drunkenness is not a national vice, it is at least one more common in America than in most countries of Europe. Indeed in the wine districts of Europe, it may almost be said to be rare. The effects of alcohol upon the human system are singularly destructive. The stomach and the brain fall beneath its blighting influence, like tender vegetation before the frosts of autumn. Alas, the formation of this habit is often the result of our Pharisaical arrogance. The *really* moderate use of alcohol does not usually cause immediate disease, in fact, many persons become stronger and more healthy from its use, yet while we know that all who use it to any extent, are liable to use it to excess, *each individual* thinks *himself* able to indulge in moderation, to go thus far and no farther. Alas, so many find, *facilis decensus Averni:* * * * * * * *Sed revocare gradum.*

But there is another habit, more universal and more peculiarly American, if not more destructive, and just as insidious in its approaches.

Tobacco! Where is thy influence? What the magic spell by which thy victims are enthralled? Oh, for some Delilah to discover the secret of thy strength. We'd shear thee of thy power, and use thee as a chained Sampson. Sages and philosophers have reasoned upon thy seductive and mischievous influences, yet both have fallen victims to thy charms. The satirist has lampooned thee, but his ridicule has fallen harmless at thy feet. Kings have written against thee and hurled weighty anathemas at thy head, yet thou dwellest in the palaces of royalty unappalled. The pulpit has denounced thee, yet thy stains may be seen upon its sacred altars. The press has waged unremitting war upon thee, bu the result of the contest is smoke, only smoke. The Doctor advises solemnly against thy use, yet, at the same time, rolls thee as a sweet morsel under his tongue. Woman has used every artifice her prolific genius could invent to rob thee of thy charm, but woman has herself yielded to thy weird influence. All grades, all professions bow to the will of the magician. In many countries art thou worshipped, but nowhere is thy worship so universal, nowhere are thy votaries so devoted, as in America. Republican America yields herself submissively to thy absolute will, and right dearly does it cost her. She pays daily taxes of wasted brain, of shrunken muscles, of energies impaired and healthful spirits broken.

There is no cause for ill-health, in our country, so universal as the use of tobacco. It is almost universally used by our male population, and a large majority of the subjects of the habit, acknowledge its bad effects upon their health. Its effects are so gradual and insidious, and the habit is so singularly irresistible, that the victims are usually powerless to relieve themselves, even when the reason for ill health is known and acknowledged, but there are no doubt, many cases of disease caused directly or indirectly by the use of tobacco, which are attributed to other causes. It might be interesting to consider the pathological effects of the use of tobacco, but it is not our purpose to do so in this paper. Suffice it to say, the nervous system is enervated, the brain is rendered inactive and sluggish, the stomach becomes unable to fulfill its duties. The victim suffers from low spirits, ennui, nervous headache, and nervous debility. The excessive flow of saliva produced is a very serious drain upon the system. The skin is rendered yellow and dry, the gums are irritated and otherwise injured; in short, the general stamina is injured, and the individual rendered less able to resist disease.

Some of the causes which lead to the use of stimulants have been referred to, but often the cause is more deep seated.

Why do we stimulate? It is no doubt often a physical necessity caused by improper training in childhood. The American system of rearing children, is, in some respects, peculiar to this country. Probably in no country is such license given them; in no country are they so indulged; in no country are they so fed. This training gives to American children an independence and self-reliance not seen in the children of any other civilized country, but some of its effects are exceedingly unhappy. The American mother gives her toothless infant, food suitable for an adult. This produces indigestion, flatulence and colic; then stimulants are resorted to for relief, opiates, cordials, &c. As the child grows older, his food is often that which the epicure of fifty years would relish. The result of this system is readily seen; if disease of the brain and nervous system do not destroy the child, an unconquerable appetite for stimulants is formed. The opiates and cordials and high feeding of childhood, result in the tobacco and alcohol of mature years. Many a fond mother has thus dug a drunkards grave for her son.

But there are other defects in our management of children, perhaps more peculiarly American, at least more directly dependent on the character of our institutions, which result in ill health and suffering.

In America, every man is to a greater or less extent, the architect of his own fortune. Individual effort will secure the highest honors, political and social; persevering energy, coupled with prudence and economy will insure wealth. These are some of the glorious advantages of our institutions, for the result is, the whole talent of the country is developed. Genius does not fail for want of opportunity; there are but few "mute Miltons;" peculiar talents and peculiar gifts are almost sure of developement. Ambition is aroused in almost every breast. Where there is no aristocracy of birth there is of necessity an aristocracy of intellect and of wealth. The developement of mind is of course dependent upon individual effort; and where there are no unjust laws of primogeniture, wealth fluctuates. The status of society is constantly changing, the lowest strata of to-day may be the surface of to-morrow. It is then but natural that the parent should be solicitous that his child be well prepared for the contest for place and power. He is, consequently, generally too early sent to school, where an unhealthy competition is begun. If possible the ambition is aroused. He is advanced too rapidly. The little undeveloped mind is *taxed*, not exercised. Physical exercise, if freely allowed, is not encouraged. Too much time is devoted to study. Let any one—the strongest and best trained mind—study closely for four consecutive hours and lassitude and weariness are felt. In many schools, eight or nine hours of the day are devoted to study, and no attention paid to the physical education, at a time, too, when so much depends upon proper training and exercise. The bodily developement is neglected, nay often worse than neglected. We know that severe labor prevents physical growth and developement. The stock raiser does not allow his young horses to be overworked. The intelligent southern planter does not impose severe labor upon his young and growing negroes; but with the minds of our children, we are less rational. The teacher is too often a hard taskmaster. The mind is overworked and developed by an unhealthy hotbed process. What is the result of this course? Our school-rooms too often present an array of pale, sickly children—what are sometimes called intellectual looking children. Instead of the bright, ruddy, laughing faces, we find a wearied, tired-looking group, thoughtless or listless; instead of the plump, round, form we find the stooped shoulders, and angular body.

But this forcing process has even a worse effect upon the mind. The intellect may seem for a time wonderfully powerful, but it is soon worn out; like a certain system of farming adopted by some planters; large yields are for a time obtained, but by constant planting of the same crop, the land is in a few years a waste, a desert. While at school, perhaps through college, the mind seems to retain its vigor, and its power, constantly acted upon by such exciting causes, encouraged to labor by its ambitious parent, his youthful mind, fired by that dreamy, undefined longing for fame, peculiar to youth, his mind seems equal to every task and admiring friends predict for him a glorious future.

But alas, these hopes are often blighted. The mind, overworked at school, is unequal to the fierce conflicts of life; he fails to meet the expectations of his friends, he fails to obtain the victories he expected to win so easily. The genius is often surpassed by the dunce of the schoolroom. The honors of the country are not most commonly won by those who obtain the honors at college.

The causes which produce this competition, labor and care, during childhood and youth, act with tenfold strength upon men struggling for place and for power. The avenues to high social position and influence are open to all. The result is, a feverish excitement and unhealthy competition among all classes, excessive toil and undue application to business. Americans labor too assiduously; and if it is not all work it is apt to be all play; intemperance in toil, or intemperance in pleasure; the excitement of labor or the excitement of dissipation.

Man is a complex piece of mechanism; every organ requires proper exercise and proper rest. The brain is a complicated organ and requires varied occupation, as well as regular rest; one set of faculties, must not be fatigued, another set of faculties must not be disused. The so-called, lighter qualities of the mind should be exercised as well as the more grave. The qualities of the mind denominated ideality should be improved as well as the mathematical faculties. No faculty should be overworked; none disused; if they are, a healthy organization will soon cease to exist. But among Americans there are a class who make a business of pleasure, while the men of business too seldom think of recreation. The unmarried woman must travel, must visit the springs, must sing and dance, but after marriage these things are put away as follies, then comes family cares, too often unrelieved by any relaxation or recreation.

The mass of the American people are energetic, business people. The day is begun in toil, the middle of the day is passed in toil, and the shades of evening close around the American still toiling, as if under unrelenting task-masters. In European countries it is not so. It is true, the poor are often down-trodden, and oppressed; with them it requires constant labor to keep soul and body together, but with the middle and upper classes it is different. In Great Britain the hunting season almost depopulates the cities; while the wealthier classes from the rural districts have their season of pleasure and recreation in town. The women of Great Britain manage to leave their family cares, (without neglecting them) for air and exercise, every day. The business man of France is as energetic and industrious as any in the world, but at the close of business hours he forgets business, and is the gay, volatile Frenchman of our childish imagination, spending the evening socially, and usually in the open air. The methodical German, with all his desire for gain, and close attention to business, is emphatically social in his habits. But the American sacrifices every thing to his business.

The merchant toils over his accounts and his merchandize the whole day, and at night dreams of his schemes for speculation, and for increase in his business. So it is in all professions and departments of business.

This close, daily attention to business would not be so objectionable if a few weeks of recreation were indulged in two or three times a year, but among the masses of our people, this is seldom done.

These remarks apply with peculiar force to the female portion of our population. They have less variety of occupation and less recreation than the other sex.

The life of the married woman is too often a continued round of indoor drudgery, housekeeping, sewing, nursing, &c., almost make up the sum total of her existence. These are obvious duties, unobjectionable in their character, but an occasional respite from them is indispensable to health. The planter usually has a pleasant holiday, when he carries his produce to market. The merchant has variety of occupation in making his Spring and fall purchases. But the gude wife is too commonly left at home in these semi business excursions. She remains at home to nurse and to mend. While the husband speculates upon the cause of his wife's failing health and anathematizes her "hysterical spells." In the cities, shopping is resorted to as a means of amusement and recreation. This is at best a recreation of doubtful character, often injurious in its effects upon mind and heart.

Reform in these particulars will be gradual; our means of amusement must be increased; their character must be improved. The cure will not be found in the crowded theatre or ball room; an indulgence in these leads to excess, and they are beside, subject to a variety of objections. Important resources of pleasure and recreation are given us by a cultivation of the more refined qualities of the mind. A taste for the fine arts should be cultivated, an appreciation of the beauties of nature should be fostered. When Americans learn to beautify their homes, to plant trees, to cultivate fruits and flowers, they will have unfailing resources for pleasure and recreation. Then the business man will forget the perplexities of commerce, in the interest of his fruits and his flowers. Home will be a pleasant resort, a place of rest, a place of amusement and recreation. But alas, the restless, roving character of our people has, to a graeat extent, prevented this. We might copy with advantage the example of the German in this particular. In some of the German cities, soil is put upon the roofs of the houses, the very housetops are cultivated.

The little plot of ground attached to each house in our towns and cities is too often bare, or cultivated only by the hired gardner. How few homes do we find in the country beautified and adorned by the hand of refinement and taste. The planter, who might, without expense, beautify his home, and make it a place to which his children will cling, and to which they will ever look back with pleasure and regret, gives all his attention to cotton ; never plants a tree, flouts at flowers as useless, neglects his orchards, and leaves the grounds about his house a waste, or plants them in corn or cotton. No wonder there is so little home feeling among us; no wonder the youth leave the old homes as soon as they attain their majority, without one sigh of regret without one tear of parting.

